# Supplementary material for: WordCommentsAnalyzer: A windows software tool for qualitative research
Source: F1000Res. 2018 Sep 4;7:536. Originally published 2018 May 3. [Version 2] doi: 10.12688/f1000research.14819.2 (PMC6137411; doi:10.12688/f1000research.14819.2)
Supplement: Supplementary file 3 [file f1000research-7-17614-s0002.tgz › b2a79496-4ada-47c4-9682-a99ce867836d.zip!/Eight Iranian Health Journals/Journal of Education and Community Health_9.docx]

© Journal of Education and Community Health. This work is licensed under CC BY-NC 4.0 (https://creativecommons.org/licenses/by-nc/4.0/).

Volume & Issue: دوره 2 - شماره 4

Title: تبعیت از درمان دارویی و عوامل مرتبط با آن در بیماران دیابتی نوع دو

http://jech.umsha.ac.ir/article-1-157-fa.html

سابقه و هدف: سطح پایین تبعیت دارویی در بیماران مبتلا به دیابت نوع دو یکی از بزرگترین چالش‌ها در درمان و کنترل بیماری است. لذا مطالعه حاضر با هدف تعیین تبعیت از درمان دارویی و عوامل مرتبط با آن در بیماران دیابتی نوع دو  در سال 1394 طراحی و اجرا شد. مواد و روش‌ها: در مطالعه مقطعی حاضر، تعداد 300 بیمار مبتلا به دیابت نوع دو دارای پرونده در خانه‌های بهداشت شهرستان تویسرکان به طور تصادفی انتخاب شدند. ابزار پژوهش شامل پرسشنامه خودساخته مربوط به عوامل موثر بر تبعیت دارویی و پرسشنامه سنجش میزان تبعیت دارویی بود. پرسشنامه‌ها پس از تائید روایی و پایایی، توسط پرسشگر و به روش مصاحبه حضوری تکمیل گردید. جهت تجزیه و تحلیل داده‌ها از آمار توصیفی و استنباطی شامل تی مستقل، آنالیز واریانس یک‌طرفه، رگرسیون خطی ساده و چندگانه با استفاده از نرم افزار SPSS نسخه 19 استفاده گردید. یافته‌ها: 26.3 درصد بیماران مذکر و 73.7 درصد مونث بودند. 65 درصد بیماران بی‌سواد و 24 درصد دچار عوارض بیماری بودند. 59.4 درصد بیماران تبعیت دارویی ضعیف داشتند. از بین متغیرهای مورد بررسی سن، سطح تحصیلات، هزینه مراقبت‌ها و درمان، تیم مراقبت و نظام سلامت، عوامل مربوط به درمان و وضعیت بیماری، باورهای مربوط به بیماری، خودکارآمدی، نگرانی‌های مربوط به مصرف دارو با تبعیت دارویی ارتباط معنی‌داری داشتند (0.05>P). نتیجه‌گیری: مطالعه حاضر نشان داد که تبعیت دارویی بیماران مبتلا به دیابت نوع دو وضعیت مناسبی ندارد و عوامل متعدد فردی، اقتصادی و اجتماعی روی آن تاثیر‌گذار است، لذا بایستی در هنگام طراحی مداخلات آموزشی نقش این عوامل در نظر گرفته شود.

Title: بهبود و تداوم رفتار ورزشی و شاخص های تن سنجی در زنان تهرانی: کاربردی از نظریه رفتار برنامه ریزی شده

http://jech.umsha.ac.ir/article-1-169-fa.html

سابقه و هدف: میزان فعالیت جسمانی بعنوان عامل اساسی سبک زندگی سالم، در زنان کمتر از حد لازم است. مداخلات آموزش سلامت نظریه محور از جمله نظریه رفتار برنامه ریزی شده (Theory of Planned Behavior) تاثیر بسزایی در ارتقا و پایداری فعالیت بدنی دارند. هدف این مطالعه بررسی تاثیر برنامه آموزشی مبتنی بر TPB بر رفتار ورزشی و شاخص های تن سنجی در زنان ساکن در خانه های سازمانی تهران بود. مواد و روش‎ها: یک کارآزمایی تصادفی شاهددار روی 130 نفر از زنان ساکن در منازل سازمانی تهران (1393) انجام شد که بطور تصادفی و توسط نمونه گیری خوشه ای چندمرحله ای انتخاب گردیدند. جلسات آموزشی بر اساس روش های تعدیل سازه های TPB (نگرش ابزاری و عاطفی، هنجارهای انتزاعی، ادراک کنترل رفتاری، قصد و رفتار) اجرا گردید. سازه های نظریه، سطح و شدت فعالیت بدنی، شاخص توده بدنی (Body Mass Index) و نسبت دور کمر به لگن (Waist Hip Ratio)، قبل، سه و شش ماه بعد از آموزش با استفاده از SPSS16 تجزیه و تحلیل شدند. یافته ها: اجرای برنامه آموزشی موجب افزایش و تداوم میانگین سازه های TPB و میانگین رتبه سطح و شدت فعالیت جسمانی، بترتیب در سه و شش ماه بعد از مداخله در گروه آزمون گردید (0.001>P). همچنین کاهش و پایداری متوسط BMI و WHR در مقاطع زمانی مشاهده شد (0.001>P). اما در گروه کنترل هیچ تغییر معناداری در متغیرهای مذکور حاصل نشد (0.05<P). نتیجه گیری: اجرای جلسات آموزشی TPB محور موجب بهبود و تداوم رفتار ورزشی و شاخص های تن سنجی در بانوان می گردد.

Title: بررسی وضعیت انجام تست پاپ اسمیر و عوامل موثر بر آن بر اساس الگوی اعتقاد بهداشتی در زنان مراجعه کننده به مراکز بهداشتی درمانی شهر قم

http://jech.umsha.ac.ir/article-1-139-fa.html

سابقه و هدف: پاپ اسمیر یک آزمایش غربالگری برای تشخیص سرطان دهانه رحم در زنان به ظاهر سالم محسوب شده و مشخص گردیده تا 60 درصد مرگ ناشی از سرطان سرویکس با غربالگری از طریق این آزمون قابل پیشگیری می­ باشد. این مطالعه با هدف تعیین وضعیت انجام تست پاپ اسمیر و عوامل موثر بر آن بر اساس الگوی اعتقاد بهداشتی در زنان مراجعه کننده به مراکز بهداشتی درمانی شهر قم انجام شده است. مواد و روش ها: در این مطالعه مقطعی 320 نفر از زنان مراجعه کننده به مراکز بهداشتی درمانی شهر قم در سال 1393 به روش نمونه­گیری تصادفی چند مرحله­ای انتخاب شدند. جهت گردآوری داده­ها از پرسشنامه محقق­ ساخته روا و پایا استفاده شد. داده­ها توسط نرم­افزار آماری SPSS در سطح معنی­ داری کمتر از 0.05 تحلیل شد. یافته ها: 11.25 درصد بطور منظم از تست پاپ اسمیر بهره گرفته و 51.87 درصد بطور نامنظم در غربالگری سرطان دهانه رحم شرکت کرده بودند و 36.88 درصد هیچگاه از این تست استفاده نکرده بودند. نتایج نشان داد که در میانگین نمرات سازه ­های الگوی اعتقاد بهداشتی بر حسب وضعیت استفاده از تست پاپ اسمیر اختلاف معنی ­داری وجود داشت (0.05>P). نتیجه ­گیری:در این مطالعه نمونه­ ها سرطان دهانه رحم را بیماری وخیمی تلقی نمی­ کردند و حتی زنانی که خود را آسیب­ پذیر احساس می­ کردند سعی می­ نمودند آن را مخفی نگه دارند، لذا در عین اینکه حساسیت درک شده و شدت درک شده از خطر عارضه در آنان کم بود موانع بیشتری را نیز درک می­ کردند و به همین دلیل میزان استفاده از تست پاپ اسمیر نامطلوب بود.

Title: بررسی همبستگی حمایت اجتماعی و رفتارهای خود مراقبتی پیشگیری از پای دیابتی در بیماران مبتلا به زخم پای دیابتی

http://jech.umsha.ac.ir/article-1-140-fa.html

سابقه و هدف: زخم پای دیابتی به عنوان یکی از مهمترین و شایع‌ترین عوارض دیابت می‌باشد. خودمراقبتی علاوه بر کاهش بستری، می‌تواند از 85 درصد از مشکلات بیماران پیشگیری نماید. حمایت اجتماعی نیز به عنوان یک عامل روانی و محیطی بر خودمراقبتی بیماران مؤثر است. لذا این پژوهش با هدف بررسی ارتباط بین رفتارهای خودمراقبتی این بیماران و حمایت اجتماعی از آنها انجام شده است. مواد و روش‌ها: در این مطالعه توصیفی 130 بیمار مبتلا به پای دیابتی در یک دوره پنج ماهه به روش سرشماری از بیمارستان شهید مدرس ساوه انتخاب و وارد مطالعه شدند. اطلاعات از طریق پرسشنامه‌های جمعیت‌شناسی، نیازهای خودمراقبتی پیشگیری از پای دیابتی و حمایت اجتماعی واکس جمع آوری گردید. جهت تجزیه و تحلیل داده ها از نرم افزار SPSS نسخه 16 و آزمون‌های آماری اسپیرمن، تی‌مستقل و آنالیز واریانس استفاده شد. یافته‌ها: در این مطالعه ارتباط معنی‌داری بین خودمراقبتی و حمایت اجتماعی مشاهده شد (P<0.05)، به این صورت که افراد با حمایت اجتماعی بالاتر خودمراقبتی بهتری داشتند. همچنین ارتباط معنی‌داری بین میزان تحصیلات و درآمد ماهیانه با رفتارهای خودمراقبتی و نیز بین میزان تحصیلات، میزان درآمد ماهیانه و وضعیت تأهل با حمایت اجتماعی افراد دیده شد (P<0.001). نتیجه‌گیری: نتایج نشان داد که حمایت اجتماعی می‌تواند به عنوان یکی از فاکتورهای تأثیرگذار بر رفتارهای خودمراقبتی افراد باشد و با در نظر گرفتن آن در برنامه‌های آموزشی، درمانی و مراقبتی، می‌توان با بهبود وضعیت خودمراقبتی، علاوه بر کاهش هزینه‌های اقتصادی، کیفیت زندگی بیماران را نیز بهبود بخشید.

Title: تاثیر آموزش نوروفیدبک بر اضطراب و افسردگی دانش آموزان مبتلا به اختلال نارسایی توجه/ بیش فعالی

http://jech.umsha.ac.ir/article-1-182-fa.html

سابقه و هدف: از روش آموزش نوروفیدبک به عنوان یک روش درمانی نسبتا جدید برای درمان بیماری‌ها و اختلال‌ها استفاده می‌شود. این پژوهش با هدف بررسی تاثیر آموزش نوروفیدبک بر اضطراب و افسردگی دانش‌آموزان مبتلا به اختلال نارسایی توجه/ بیش فعالی انجام شد. مواد و روش‌ها: پژوهش حاضر یک مطالعه نیمه تجربی از نوع پیش‌آزمون و پس‌آزمون همراه با گروه کنترل بود. جامعه آماری شامل همه دانش‌آموزان ابتدایی مبتلا به اختلال نارسایی توجه/بیش فعالی مراجعه کننده به مراکز مشاوره شهر پاکدشت در سال 1394 بودند. در مجموع 40 دانش‌آموز با روش نمونه‌گیری در دسترس انتخاب و به طور تصادفی در دو گروه (هر گروه 20 نفر) جایگزین شدند. گروه‌ آزمایش 12 جلسه 60 دقیقه‌ای با روش‌ نوروفیدبک آموزش دیدند. برای جمع‌آوری داده‌ها از پرسشنامه‌های اضطراب کتل و افسردگی بک استفاده شد. داده‌ها با استفاده از نسخه 19 نرم‌افزار SPSS و با روش کوواریانس چند متغیری (MANCOVA) تحلیل شدند. یافته‌ها: نتایج نشان داد که قبل از مداخله میانگین و انحراف معیار اضطراب و افسردگی در گروه نوروفیدبک به ترتیب 4.22±46.63 و 5.83±37.61 بود، اما پس از مداخله میانگین و انحراف معیار اضطراب و افسردگی در گروه نوروفیدبک به ترتیب به 3.81±35.09 و 3.64±25.78 تغییر یافت. همچنین نتایج نشان داد که روش آموزش نوروفیدبک به طور معنی‌داری باعث کاهش اضطراب و افسردگی دانش‌آموزان مبتلا به اختلال نارسایی توجه/ بیش فعالی گردید (0.001>P). نتیجه‌گیری: با توجه به نتایج این پژوهش پیشنهاد می‌شود درمانگران و روانشناسان بالینی از روش آموزش نوروفیدبک برای کاهش اضطراب و افسردگی دانش‌آموزان مبتلا به اختلال نارسایی توجه/ بیش فعالی استفاده نمایند.

Title: بررسی تاثیر مداخله آموزشی مبتنی بر الگوی پرسید-پروسید بر ارتقای رفتارهای ایمن ترافیکی دانش آموزان دختر مدارس ابتدایی شهر تبریز در سال 1393

http://jech.umsha.ac.ir/article-1-156-fa.html

سابقه و هدف: آسیب‌های عابران پیاده از معضلات مهم بهداشت عمومی بوده و یکی از قربانیان عمده آسیب‌های ترافیکی کودکان می‌باشند. آموزش یکی از مهم‌ترین استراتژی‌های رفع آسیب‌های ترافیکی است، ولیکن جهت کسب نتایج مفید و مؤثر، آموزش می‌بایست بر مبنای نظریه‌ها و الگوهای تغییر رفتار طراحی گردد. بنابراین، هدف مطالعه حاضر تعیین تاثیر مداخله آموزشی مبتنی بر الگوی پرسید-پروسید بر ارتقای رفتارهای ایمن ترافیکی دانش آموزان دختر مدارس ابتدایی شهر تبریز بود. مواد و روش‌ها: پژوهش حاضر یک مطالعه کارآزمایی شاهددار تصادفی بود. جامعه پژوهش شامل 75 دانش آموز پایه چهارم ابتدایی از 2 مدرسه دولتی دخترانه بود که به صورت تصادفی انتخاب شدند. دانش آموزان پرسشنامه مبتنی بر الگوی پرسید-پروسید را قبل و یک ماه بعد از مداخله آموزشی تکمیل نمودند. مداخله شامل 6 جلسه آموزشی بود که در مدارس گروه آزمون اجرا گردید. یافته‌ها در نسخه 18 نرم افزار SPSS و با استفاده از آزمون‌های فیشر، کای اسکوئر، تی‌مستقل و تی‌زوجی تحلیل و بررسی گردید. یافته‌ها: میانگین سنی شرکت کنندگان در پژوهش 0.68±10.11 بود. تفاوت معنی‌داری در خصوص متغیرهای دموگرافیک قبل از مداخله آموزشی بین دو گروه مشاهده نشد (P>0.05). نتایج نشان دهنده وجود تفاوت معنی‌دار در میانگین نمرات آگاهی، نگرش، عوامل مستعد کننده، عوامل قادر کننده، عوامل تقویت کننده و عملکرد در گروه آزمون پس از مداخله آموزشی نسبت به گروه کنترل بود (P<0.05). نتیجه‌گیری: مداخله آموزشی بر اساس الگوی پرسید-پروسید می‌تواند سبب ارتقای رفتارهای ایمن ترافیکی دانش آموزان گردد.

Title: میزان استرس زندگی دانشجویی و عوامل مرتبط با آن در دانشجویان دانشگاه علوم پزشکی همدان در سال 1394

http://jech.umsha.ac.ir/article-1-199-fa.html

سابقه و هدف: استرس در دوران زندگی دانشجویی می‌توانند منجر به پیامدهای منفی متفاوتی از قبیل بیماری‌های جسمی، اختلالات روانی یا احساس فرسودگی در دانشجویان گردد. لذا مطالعه حاضر با هدف تعیین میزان استرس زندگی دانشجویی و عوامل مرتبط با آن در دانشجویان دانشگاه علوم پزشکی همدان انجام گردید. مواد و روش ها: ﭘﮋﻭﻫﺶ ﺣﺎﺿﺮ ﻳﻚ ﻣﻄﺎﻟﻌﻪ ﻣﻘﻄﻌﻲ است ﻛﻪ بر روی 500 نفر از دانشجویان که به‌صورت تصادفی چندمرحله‌ای انتخاب شده بودند، انجام گردید. پرسشنامه مشتمل بر دو بخش مشخصات دموگرافیک و پرسشنامه استاندارد استرس دوران زندگی دانشجویی بود که به روش خودگزارش‌دهی تکمیل گردید. داده‌ها با استفاده از ویرایش 20 نرم‌افزار SPSS و بهره‌گیری از آزمون‌های آماری تی‌مستقل، آنالیز واریانس یک‌طرفه و ضریب همبستگی پیرسون تجزیه و تحلیل گردید. یافته‌ها: طبق یافته‌ها 57 درصد از دانشجویان نمره استرس متوسط داشتند. بزرگ‌ترین استرسورهای تجربه شده در درجه نخست استرس خود تحمیلی و سپس فشارها می‌باشد و بیشترین واکنش به این عوامل در درجه اول واکنش شناختی و سپس واکنش هیجانی بوده است. از بین متغیرهای جمعیت‌شناختی رشته تحصیلی، مقطع تحصیلی، تحصیلات مادر و تحصیلات پدر با استرس دوران دانشجویی رابطه معنی‌داری داشتند (P<0.05). نتیجه‌گیری: با توجه به سطح بالای استرس در دانشجویان و مشخص شدن عوامل مؤثر جمعیت‌شناختی می‌توان نسبت به مداخله مؤثر در جهت مساعد کردن این عوامل اقدام کرد.
